# The effect of different salivary calcium concentrations on the erosion protection conferred by the salivary pellicle

**DOI:** 10.1038/s41598-017-13367-3

**Published:** 2017-10-11

**Authors:** T. Baumann, R. Bereiter, A. Lussi, T. S. Carvalho

**Affiliations:** Department of Preventive, Restorative and Pediatric Dentistry, University of Bern, Freiburgstrasse 7, CH-3010 Bern, Switzerland

## Abstract

Different proportions of mineral ions in saliva can influence the protective effect the salivary pellicle provides against dental erosion. To investigate the effect of different calcium concentrations in human saliva on the protection against enamel erosion, enamel specimens were divided into 8 treatment groups: humid chamber (Ctrl); whole mouth stimulated human saliva (HS); artificial saliva containing different calcium concentrations (AS_low_, AS_medium_, AS_high_); and dialysed human saliva containing different calcium concentrations (DS_low_, DS_medium_, DS_high_). The specimens underwent 4 cycles of incubation in the treatment group followed by an erosive challenge. Surface hardness and calcium release were measured during the cycling process. All DS groups exhibited significantly higher enamel surface softening than HS and the corresponding AS groups. Among the DS groups, the surface softening was significantly higher in DS_low_ than in DS_high_. No significant differences were found within the AS or DS groups regarding calcium release. The results of this study indicated that erosion protection conferred by saliva depends on an interplay between salivary proteins and ions. While both proteins and ions have a positive effect on their own, the combination of the two can lead to different degrees of protection or even negative effects, depending on the relative concentrations.

## Introduction

Erosive tooth wear (ETW) is described as the chemical-mechanical process resulting in a cumulative loss of hard dental tissue not caused by bacteria^1^. As Jaeggi *et al*. reported, it is a common condition in developed societies with a prevalence ranging from 4% to 100% in adults^2^. ETW begins with an initial demineralization (surface softening) of the enamel caused by the chemical action of extrinsic or intrinsic acids^3^.

Human saliva is an important natural factor that protects against erosive demineralisation. Apart from the activity of human saliva in diluting, clearing, neutralizing and buffering acids, it also reduces demineralization and enhances the remineralization process. The most effective ions present in human saliva that play a role in this protection are calcium, phosphate and fluoride^4^. In addition to the ions, saliva also protects from acidic challenges by forming a salivary pellicle on the tooth surface.

Salivary pellicle formation starts with the adsorption of single peptides and proteins onto the enamel surface; within one minute, an electron dense pellicle layer of 10–20 nm thickness can already be observed^5^. Pellicle formation carries on as a selective process, leading to the formation of two salivary pellicle layers^5^. The first (basal) pellicle layer is formed with phosphate- and calcium-binding peptides and proteins, especially statherins, histatins and acidic proline-rich proteins. The second layer is formed from heterogeneous protein accumulation and also contains other biomacromolecules^6,7^. The calcium-binding peptides present in the basal pellicle layer can bind free calcium ions from the surrounding saliva and act as a calcium reservoir in the salivary pellicle, thus allowing mineral homeostasis on the enamel surface^6^. Furthermore, calcium can diffuse easily through the pellicle, and this calcium exchange between saliva and the tooth surface is important for the remineralization processes^8^.

Previous studies have discussed the effect of salivary components on erosive demineralisation. Lussi *et al*. showed that, in contrast to healthy individuals, patients with erosive demineralisation present a lower pH drop after an erosive attack and a reduced ability to reach initial pH conditions^9^. This could be related to the lower buffer capacity in saliva from patients with ETW. Moazzez *et al*. later discussed that salivary pellicles from patients with ETW were probably different from pellicles from healthy patients; the latter exhibited a better protective effect for enamel^10^. In a subsequent study from the same group, Carpenter *et al*. went on to show specific differences in the composition of salivary pellicles between the two groups^11^. Newly formed salivary pellicles from patients with ETW presented less total protein, a reduced amount of statherin (calcium-binding protein), and a reduced amount of calcium^8^.

In a recent study, Baumann *et al*. showed the effect of proteins and of the interaction between proteins and mineral ions on the protective effect of salivary pellicles, and the authors concluded that different components in saliva have different protective effects^12^. More specifically, the right proportions between salivary proteins and mineral ions are critical for the ability to form a salivary pellicle that can better protect against erosive demineralisation. Because the protective effect of saliva is not related purely to the calcium concentration or protein content in saliva but rather to the association between these two factors, the present study seeks to investigate the effect of human saliva with different calcium concentrations on the protection against dental erosion.

## Materials and Methods

### Specimen preparation

A total of 80 specimens were prepared from human molars, which were selected from a pool of extracted teeth. The teeth were extracted by dental practitioners in Switzerland (no water fluoridation, 250 ppm F^−^ in table salt) and were stored in 2% chloramine T trihydrate solution. The experiment was carried out in accordance with the approved guidelines and regulations. The patients were informed about the use of their teeth and consent was obtained. Because we were using teeth from a pool of extracted teeth, the local ethics committee (Kantonale Ethikkommission: KEK) categorized them as “irreversibly anonymised”, and therefore no ethical approval was necessary.

Using an Isomet^®^ low speed saw (Isomet, Buehler Ltd., Düsseldorf, Germany) the roots were removed and the crowns were cut into buccal and oral halves. The enamel surfaces were coated with nail varnish and embedded in acrylic resin (Paladur^®^; Heraeus Kulzer, Hanau, Germany). The specimens were then ground and polished with abrasive silicon carbide paper discs of grain size 18.3 µm, 10 µm, 5–6 µm and 3 µm for 60 s each. Between the grinding and polishing steps, the slabs were rinsed and sonicated for 1 min. During this grinding/polishing process, 200 µm of the surface enamel was removed.

The polished enamel specimens were stored in a mineral solution (1.5 mmol/l CaCl_2_, 1.0 mmol/l KH_2_PO_4_, 50 mmol/l NaCl; pH 7)^13^, and immediately before to the start of the experiment, the specimens were further polished with a 1 µm diamond abrasive for 60 s under constant cooling and sonicated for 1 min.

### Incubation environments

The specimens were randomly distributed into 8 treatment groups (n = 10, Table 1): humid chamber (control, Ctrl group); human whole stimulated centrifuged saliva (HS group); three artificial saliva (AS) groups (AS_low_, AS_medium_, and AS_high_); and three groups of dialysed HS (DS), which were prepared by dialysing HS with the different AS groups (DS_low_, DS_medium_, and DS_high_) (Table 1).

**Table 1:** Calcium, phosphate and total protein concentrations in each incubation solution according to the different experimental groups.

Group	Solution	Dialysis Method	Calcium (mmol/l)	Phosphate (mmol/l)	Total Protein (µg/ml)
HS	Human saliva	—	1.11	3.46	932.96
AS_low_	Artificial saliva, low calcium	—	0.57	0.86	—
AS_medium_	Artificial saliva, medium calcium	—	0.98	0.85	—
AS_high_	Artificial saliva, high calcium	—	2.05	0.87	—
DS_low_	Human saliva, low calcium	AS_low_	0.46	0.73	717.16
DS_medium_	Human saliva, medium calcium	AS_medium_	0.91	0.7	751.32
DS_high_	Human saliva, high calcium	AS_high_	1.85	0.74	742.87
Ctrl	Control group	—	—	—	—

The 3 types of artificial saliva (AS) had the same general composition (0.9 mM KH_2_PO_4,_ 130 mM KCl, 60 mM Tris, pH 7.4) except for their calcium concentrations: AS_low_ contained 0.5 mM Ca(NO_3_)_2_, AS_medium_ 1.0 mM Ca(NO_3_)_2_, and AS_high_ 2.0 mM Ca(NO_3_)_2_.

The DS groups had similar total protein concentrations but different calcium concentrations as a result of the dialysis with the different AS (Table 1).

### Experimental design of the study

This study consisted of a cyclic experiment of incubations of the specimens in the different kinds of saliva (or humid chamber) and erosive challenges in citric acid. Initially, the surface hardness (SH) of all the specimens was measured and labelled SH_0_. They were then individually incubated in 1.8 ml of the respective incubation environment (according to the group) in a shaking water bath at 37 °C for 60 min (70 rpm, travel path 22 mm, GFL Gesellschaft für Labortechnik mbH, Burgwedel, Germany). Afterwards, the specimens were removed from the solution and rinsed with deionised water for 20 s, air dried for 5 s, and SH was measured.

The specimens were then submitted to an erosive challenge, consisting of individually immersing the specimens into 10 ml of citric acid (1%; pH 3.6) at 25 °C for 1 min (70 rpm, travel path 15 mm, P-D Industriegesellschaft mbH, Prüfungswerk Dresden, Germany). After this, the specimens were rinsed (20 s), dried (5 s) and SH was measured again. The citric acid solutions were labelled and stored for later calcium analyses.

This procedure was then repeated for a total of 4 cycles (4 incubation periods in the respective environment/solution, and a total of 4 min of erosion). SH was measured at different times: at baseline (SH_0_), after each incubation in the respective incubation environment (odd numbered values: SH_1_, SH_3_, SH_5_, and SH_7_), and after each erosion challenge (even numbered values: SH_2_, SH_4_, SH_6_ and SH_8_). The examiner performing the measurements was blinded to the identity of the solutions.

### Saliva collection

The donors of the whole mouth stimulated human saliva (HS) were informed not to eat or drink anything (apart from water) for 2 h before saliva collection. Adult participants chewed for 10 min on paraffin wax and collected the stimulated saliva in a chilled vial. The saliva was directly pooled, centrifuged at 4 °C for 20 min (4000 g), and the supernatant was divided into aliquots of 20 ml and stored at −80 °C. The donors provided their informed oral consent to use the saliva for research purposes in this study. No ethical approval was necessary because the saliva pool was categorized as “irreversibly anonymised”, and the experiment was carried out in accordance with the approved guidelines and regulations of the local ethics committee (Kantonale Ethikkommission: KEK).

### Dialysis of human saliva against different artificial saliva solutions

For the dialysed human saliva groups (DS_low_, DS_medium_, and DS_high_), aliquots of 20 ml of HS were thawed and dialysed in 2 l of the respective AS (AS_low_, AS_medium_, and AS_high_) for a total of 48 h at 4 °C using a Mega Pur-A-Lyzer^TM^ Dialysis Kit with a membrane cut-off of 1 kDa (PURG10020, Sigma-Aldrich, Switzerland). AS was exchanged after 2, 8, 16 and 24 h. After dialysis, the solutions were stored in aliquots of 1.85 ml at −80 °C until the time of the experiment.

### Characterization of the solutions

The total calcium concentration in all solutions was determined using an atomic absorption spectrometer (AAS; AAnalyst 400, Perkin Elmer Analytical Instruments, Waltham, MA, USA). Lanthanum nitrate (0.5%, lanthanum nitrate hexahydrate: La(NO_3_)_3_·6H_2_O) was added to the solution to eliminate the interference of other ions^14^.

The inorganic phosphate concentration was analysed photometrically using the method reported by Chen *et al*.^15^. From the test solution, an aliquot was diluted with ultra-pure water, and 2 ml of this dilution was mixed with 2 ml of a phosphate reagent (2% ascorbic acid, 0.5% ammonium heptamolybdate, 0.6 M H_2_SO_4_). This mixture was stored for 90 min at 37 °C, allowed to cool to 24 °C, and then absorbance was measured at 820 nm using a spectrophotometer.

The total protein concentration was determined colorimetrically using a Pierce^TM^ BCA Protein Assay Kit (Thermo Scientific) with bovine serum albumin (BSA) as a standard. The assay was performed in a 96-well micro plate using triplicates of 25 µl of each sample and standard. The plates were read at 570 nm using an ELx808 Absorbance Reader (BioTek).

### Surface hardness measurement

Vickers hardness numbers (VHN) were determined using a Vickers diamond under a pressure of 50 mN for 15 s (Fischerscope HM2000 XYp; Helmut Fischer, Hünenberg, Switzerland). For each SH measurement, seven indentations were made in a line at an overall distance of 200 μm on the enamel surface. The specimens were always placed in the same position on the device for further measurements, which were performed at 100 μm intervals from the previous measurement. The average value from the seven indentations at each step was considered for analysis. The changes in enamel hardness between the initial measurement and the following measurements were calculated as a percentage (%SH) and used for statistical data analysis and interpretation. %SH was calculated using the formula: %SH = (SH_i_/SH_0_) × 100, where SH_0_ is the initial hardness value and SH_i_ is the hardness value of the i^th^ measurement (after the i^th^ incubation in solution or after the i^th^ erosion).

### Enamel surface area measurement and calcium release

An image of the exposed enamel of each specimen was recorded with a light microscope (Leica M420 equipped with a Leica DFC495 camera). The software program IM500 was used to manually outline and automatically calculate the area of the exposed enamel.

The amount of calcium released after every single erosive challenge was determined as described above. The measured amount of calcium was normalized to the corresponding calculated enamel surface area.

### Statistical analysis

All statistical results were calculated with R 3.2.2 (R Project for Statistical Computing, Vienna, Austria), and the level of significance was set to 0.05. First, the assumption of normally distributed data was checked using graphical methods and Shapiro Wilk’s test. For both outcomes SH and calcium release, normality was rejected (p < 0.0001). The main effects of the whole-plot factor group and of time and their interaction were then tested using a non-parametric time-related ANOVA^16^. The resulting p values were corrected for multiple testing with Holm’s method. In the case of significance in the global test, a post-hoc analysis was performed with Kruskal-Wallis tests for simultaneously comparing all of the different groups and then with Wilcoxon-Mann-Whitney tests for pair-wise comparisons. P values in this section were not corrected for multiple testing because of the explorative nature of this part of the analysis.

### Data availability

The datasets generated during and/or analysed during the current study are available from the corresponding author on reasonable request.

## Results

### Surface hardness (SH)

**Figure 1:**
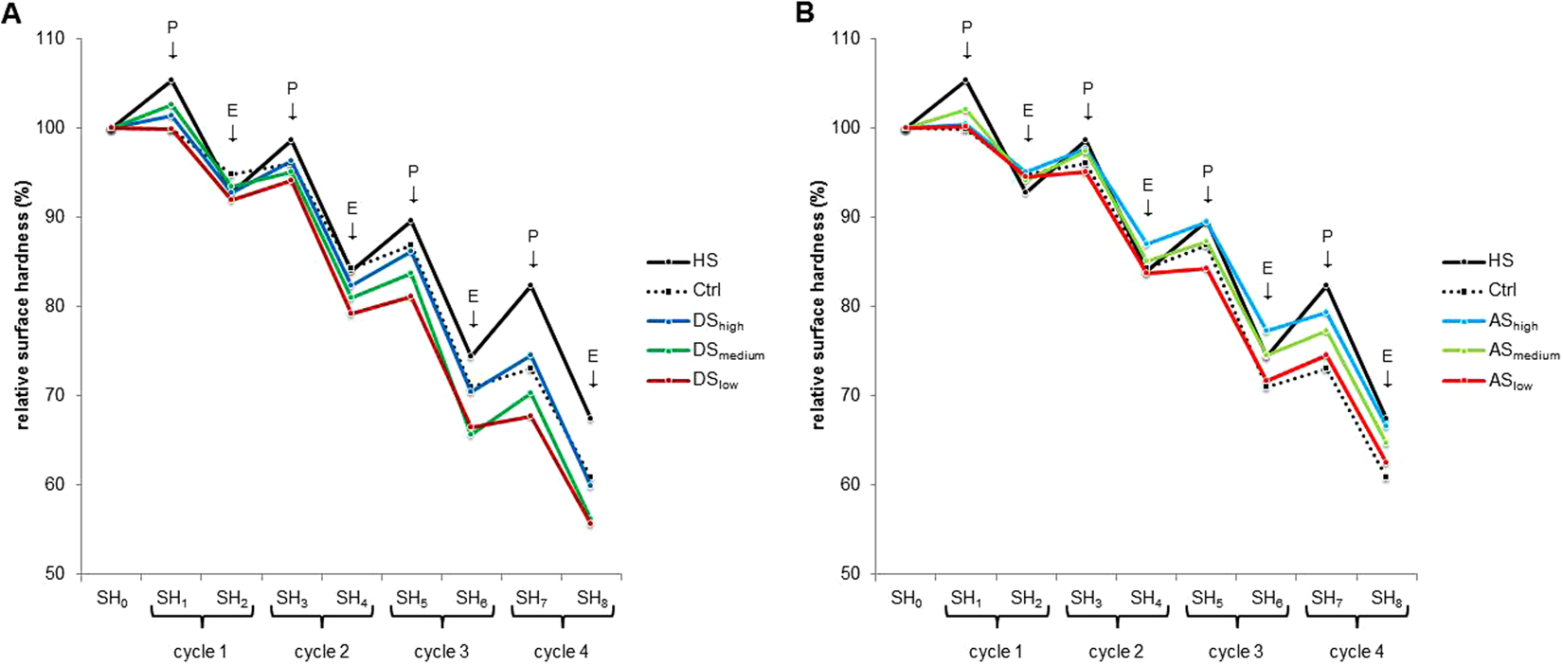
Change in mean relative SH during incubation-erosion cycles. (**A)** SH of all DS groups compared to HS and Ctrl. **(B)** SH of all AS groups compared to HS and Ctrl. P marks measurements after saliva incubation periods; E marks measurements after erosions.

SH was measured nine times: initially (SH_0_) and after each subsequent incubation and erosion treatment (SH_1–8_). The mean ± standard deviation of SH_0_ for all specimens was 519.2 ± 38.7 VHN. Figure 1A and B show the mean values of the different groups throughout the entire experimental time. Enamel softening occurred in all groups as the experiment progressed, but SH values behaved differently for different groups. In general, we observed that incubation in the different solutions led to an increase in SH. This increase in SH was most evident in the HS group. Despite the increase, the initial SH values before erosion treatments could not be restored, as the decrease in mean SH values after each erosive attack was greater than the increase during the next incubation period. This phenomenon was not only evident in HS but also in all of the other groups.

**Figure 2:**
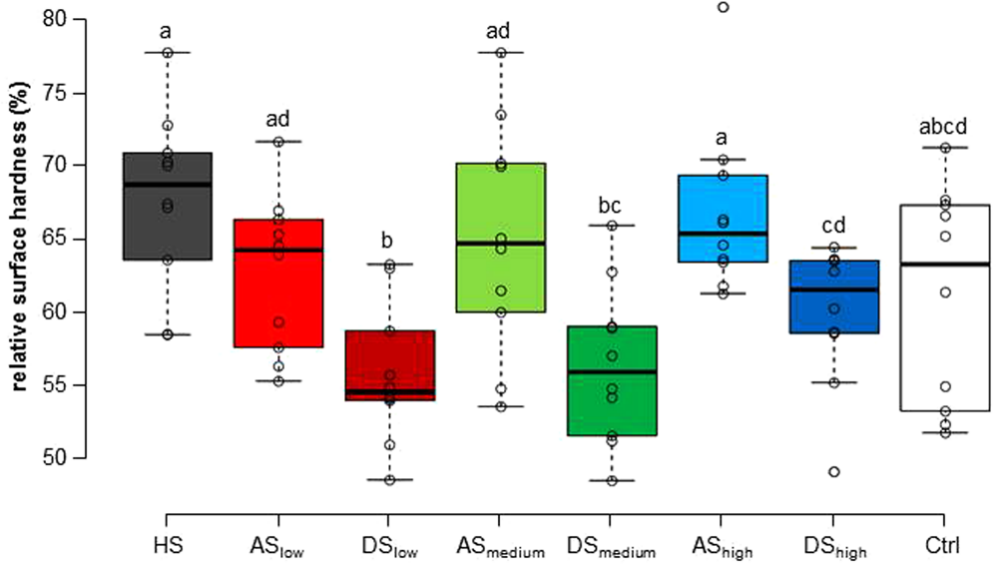
Final remaining relative SH after all experimental cycles for the different groups. SH decreased significantly less in the AS groups than in the corresponding DS groups, and SH decreased significantly less in the HS group than in all of the DS groups. Different letters indicate significant differences between the groups (Wilcoxon-Mann-Whitney test, p < 0.05).

The overall analyses showed a significant effect of group from the third incubation period onward (SH_5_; p < 0.01). The between-group differences were the most pronounced after the last cycle (SH_8_, Fig. 2). The HS group decreased from 100% to an average of 66.7 ± 5.8%, which was a significantly lower decrease than the three groups incubated in dialysed saliva (p < 0.02). The SH in the three dialysed saliva groups (Fig. 1A) decreased from 100% to 55.7%, 56.3% and 60.2% for DS_low_, DS_medium_, and DS_high_, respectively. Comparing DS_low_ with DS_high_ also showed significantly different values, where DS_low_ presented a greater SH decrease (from 100% to 55.7 ± 4.7%) in comparison to DS_high_ (decrease from 100% to 60.2 ± 4.8%). By contrast, there were no significant differences between the HS and the three groups incubated in AS (p > 0.05). Among the AS groups, there was a trend in which higher calcium concentrations led to a lower SH decrease, but the differences were not significant (Fig. 1B). Furthermore, the results show that each dialysed saliva group is significantly different from the artificial saliva with which it was dialysed (p < 0.02), in that the enamel specimens incubated with the different kinds of dialysed saliva had generally lower SH values then the specimens incubated in the corresponding artificial saliva (Fig. 2). The SH value of Ctrl at the final measurement exhibited a decrease to 61.1 ± 7.4%, which was not significantly different from any of the other groups.

### Calcium release during erosion

**Figure 3:**
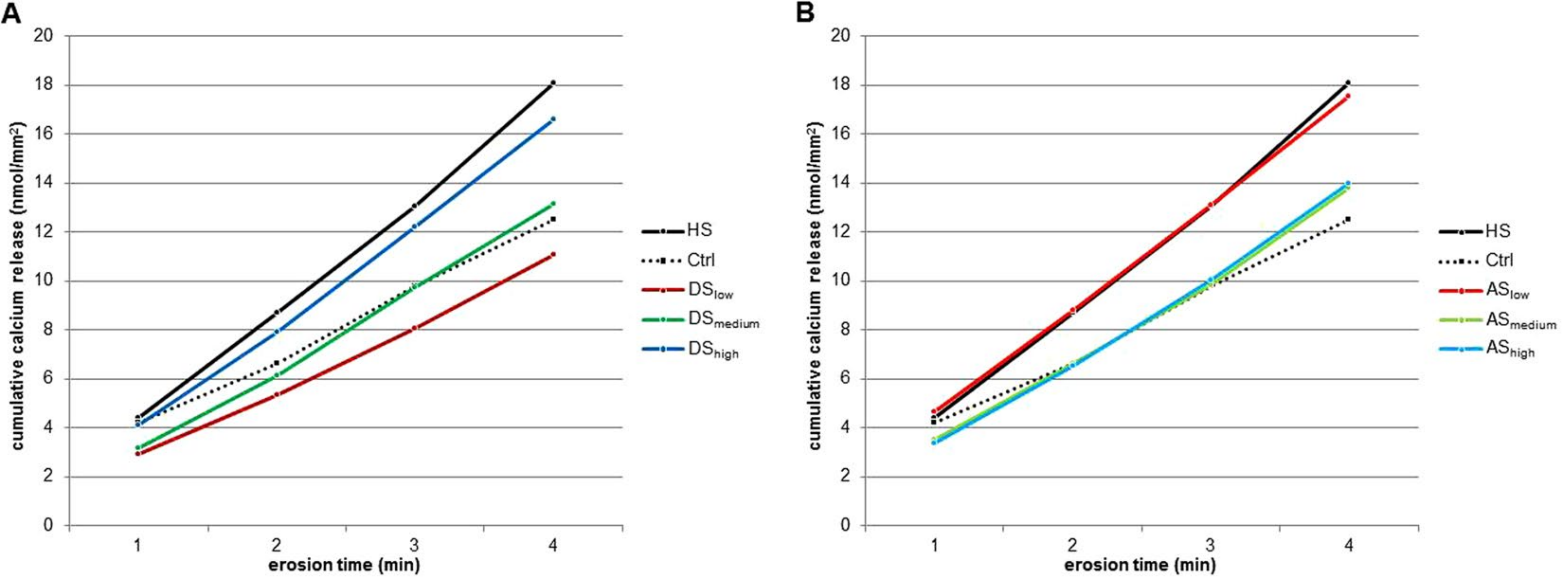
Mean cumulative calcium released during erosion cycles. **(A)** Calcium released for all DS groups compared to HS and Ctrl. A trend toward more calcium release from DS_low_ to DS_high_ was observed. **(B)** Calcium released for all AS groups compared to HS and Ctrl. A trend toward less calcium release from AS_low_ to AS_high_ was observed.

**Figure 4:**
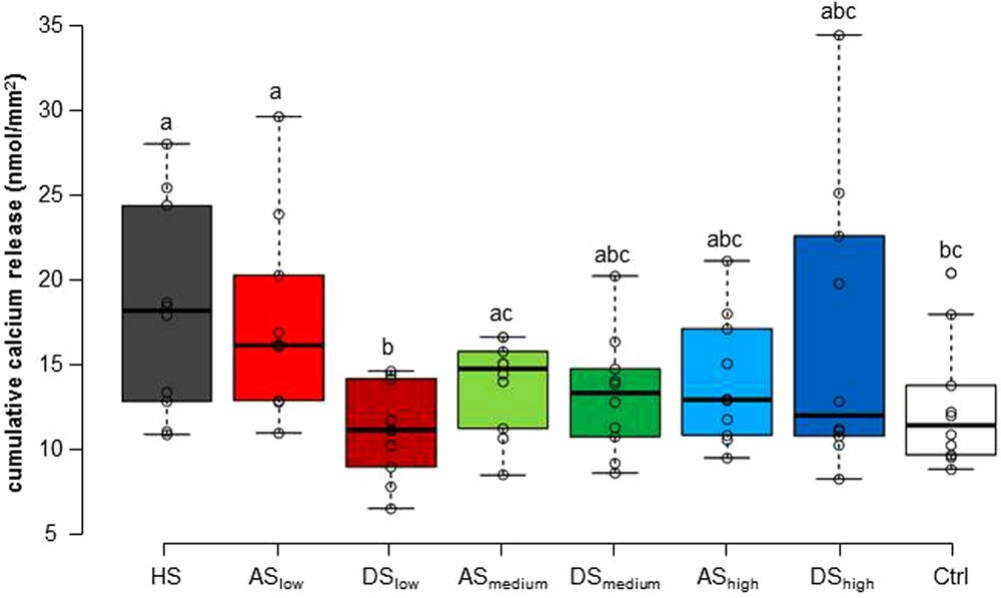
Total amount of calcium released after all experimental cycles for the different groups. No significant differences were observed within the DS and the AS groups. Different letters indicate significant differences between the groups (Wilcoxon-Mann-Whitney test, p < 0.05).

Calcium release was measured after each erosive challenge. Figure 3 presents the cumulative calcium released throughout the entire experiment. As expected, all groups released more calcium as the experiment progressed. Figure 4 shows the total amount of calcium released. Overall, the HS group released the most calcium, and some significant differences were observed, but not within the AS or the DS groups.

Within the AS groups, a clear trend was observed in which AS_low_ released more calcium than AS_medium_ and AS_high_, which both released similar amounts (Fig. 3B). However, the differences between the AS groups were not significant. Within the DS groups, the trend observed was the opposite: DS_high_ released the most calcium, followed by DS_medium_ and DS_low_ which released the least calcium (Fig. 3A). The trend was consistently observed in all cycles, although again the differences between the DS groups were not significant with respect to total calcium released (Fig. 4). Comparing each AS group with its corresponding DS group, AS_low_ released significantly more calcium than DS_low_ (p < 0.02). The other two AS groups did not differ significantly from their corresponding DS groups.

## Discussion

The mature salivary pellicle acts as a semipermeable membrane and as a diffusion barrier^17^. However the protective potential of saliva is limited and there are several individual differences, such as saliva compositions, flow rate or the pH value^11^. A common clinical indicator of the saliva properties is the flow rate of unstimulated and stimulated saliva and their pH value^18^. The average unstimulated salivary flow rate is approximately 0.35 ml/min, whereas the flow rate for stimulated saliva is reported to be approximately 2 ml/min^19^. However, one should be aware that the salivary parameters of stimulated and unstimulated saliva are different^11^. For unstimulated saliva the calcium concentration is lower than for stimulated saliva, for the protein concentration it is the opposite. To overcome individual differences and obtain an “average” saliva, we used pooled saliva from many different donors^20^.

We prepared the DS groups by using dialysis membranes with a cut-off of 1 kDa. While this retained most of the proteins, saliva also contains peptides that are smaller than 1 kDa. These peptides may have been removed by dialysis, resulting in the overall lower protein concentration of the DS groups compared to the HS group (Table 1). However, the protein concentration among the DS groups was the same, and a minor removal of peptides was tolerated as pellicle formation is a specific process.

The SH results for the AS groups showed that higher calcium concentrations led to higher SH values. Higher calcium concentrations mean that the AS is more supersaturated with respect to enamel, so more hydroxyapatite (HAP) crystals will form on the surface. This leads to higher SH values after the incubation periods (odd numbered SH values). These crystals then provide some protection for the enamel surface during the ensuing erosion, resulting in higher SH values after erosion as well (even numbered SH values, Fig. 1B). Although the differences in the end were not significant, the trend could be observed through all the cycles. A similar trend could be observed for the DS groups (Fig. 1A). The DS_high_ group showed the highest SH values, followed by DS_medium_ and DS_low_, which showed rather similar values. At the end of the cycles, DS_high_ was significantly different from DS_low_. This difference cannot be attributed solely to differences in the concentrations of ions, as the difference between the AS groups was not significant. Neither can it be explained by differences in the protein composition, as all DS groups were treated the same way and contained similar amounts of proteins (Table 1). Hence, the combination of proteins and ion concentrations has to be responsible for the observed differences. When comparing the DS with the corresponding AS groups, it was noticeable that the AS groups consistently exhibited higher SH values, with the differences being significant after the last cycle (Fig. 2). This behaviour has been observed before^12^ and is in contrast to the HS group, which showed the highest SH values of all groups. While both mineral ions and salivary proteins on their own^12^ confer protection to the enamel surface, this does not always appear to be the case for mixtures of the two. In solutions containing proteins, the balance of enamel porosity, the degree of saturation of the solution with respect to enamel minerals, and the concentrations of proteins in the solution all influence the effect the solution has on the enamel surface^21^. Faster absorption of proteins to eroded enamel has been reported^22^, while different concentrations of proteins showed varying effects on the dissolution kinetics of porous hydroxyapatite^21^. Furthermore, the influence of proteins on enamel demineralization varies significantly depending on how much is adsorbed and how much free calcium is available near the surface^23^. Finally, different proteins can either promote or inhibit erosion of enamel or lesion remineralization^21,24,25^. These examples illustrate that enamel de- and remineralization in solutions containing different proteins as well as mineral ions is very complex, with slight alterations of one factor possibly changing the effect of the solution. In the present study, while the AS and DS groups contained the same concentrations and species of ions, HS also contained other ions. Moreover, some small peptides might have been removed from the DS groups by dialysis, as the molecular weight cut-off of the membrane of 1 kDa could not retain all peptides (Table 1). The different peptide and ion concentrations between HS and DS and the additional ions present in HS might be responsible for the differences observed between these groups, with HS being significantly different from all DS groups at the end. For instance, Na^+^ has been proposed to influence the dissolution of enamel, likely through the competition for surface protonation sites between Na^+^ and H^+^ ions^18^. Furthermore, Mg^2+^ has been shown to reduce the rate of precipitation of HAP, probably by adsorbing to growth sites at the crystal surface^26^. The difference between the AS and DS groups can be explained by the presence of calcium- and phosphate-binding proteins in saliva. These proteins partly “remove” the available ions, possibly even rendering the solution undersaturated with respect to enamel^21^. Undersaturated solutions can further demineralize enamel, which could also explain the lower SH values found in the DS_medium_ and DS_low_ groups compared to the Ctrl group.

In contrast to other studies, the HS group was not significantly different from the Ctrl group at the end (Fig. 2). This can be attributed to the rather small number of samples per group (n = 10). The patterns observed in the decrease of SH, as well as the average values of these groups, are similar to earlier studies, implying that the differences would indeed be significant if the sample sizes were larger.

Calcium release is usually a sensitive method for measuring the amount of calcium released from the surface of enamel during erosion^14^. In our case, however, calcium did not arise only from the enamel surface, but also from mineral deposits (HAP crystals) formed on the enamel surface and from calcium bound by the pellicle. Nevertheless, with a careful analysis of the results, it is still possible to draw some conclusions. HAP crystals were able to form because all of the saliva used here was supersaturated with respect to pure HAP as well as enamel^27^. Although only approximately 2% of the surface of the specimens was enamel, the rest being resin, HAP crystals formed preferentially on the exposed enamel surface because there were already nucleation sites present, whereas on resin a new nucleation would be required. Therefore, it can be assumed that all the calcium released during erosion came from the enamel itself or deposits on the enamel surface but not from deposits on the resin surface. In the case of the pellicle, however, it is different. Pellicles formed not only on the enamel surface but on the whole surface of the specimen. Differences in composition, and therefore also calcium content, of the pellicles on the surfaces of enamel and resin are very likely, as the surface properties differ considerably. During erosion, calcium is released not only from the enamel surface, but also from the pellicle itself, and it is impossible to distinguish between the amounts released from these different origins. It is therefore very difficult to draw conclusions about the erosion protection conferred by these groups from the calcium released by the groups containing proteins (HS and DS groups).

From the AS groups, AS_low_ released significantly more calcium than the Ctrl group, whereas the AS_medium_ and AS_high_ groups released an amount of calcium similar to the Ctrl group (Fig. 3B). Few HAP deposits would have been formed in the AS_low_ group because it contained only a small amount of calcium and was only slightly supersaturated with respect to enamel. During the erosion cycles, the small number of deposits were not able to provide significant protection for the enamel, and they were also dissolved themselves. The calcium released from the enamel and the additional calcium released from the deposits resulted in an overall higher calcium release than in the Ctrl group. The AS_medium_ and AS_high_ groups could form larger amounts of deposits on the enamel surface because they were more supersaturated with respect to enamel. Upon the erosive attack, these deposits could provide a certain amount of protection for the enamel, and they were also dissolved themselves. This resulted in a total calcium release similar to unprotected enamel (Ctrl).

For the above-described reasons, it is not possible to draw conclusions from our experimental setup regarding the enamel-protective properties of the pellicles formed by the DS groups. But it is obvious that the pellicle itself stores calcium and releases this calcium during erosive cycles. Although the cumulative calcium release did not differ significantly between the DS groups, a clear and consistent trend was observed that DS_high_ released the most calcium, followed by DS_medium_, which released intermediate amounts, and DS_low_, which released the least amount of calcium (Fig. 3A). For DS_low_, the cumulative amount of released calcium was even lower than the Ctrl group. This hints at a protective effect for the enamel, although the difference was not significant. A similar protective effect has previously been observed for dialysed saliva containing only trace amounts of calcium and suggests strong protection of the enamel by salivary proteins^12^.

Additionally to SH and calcium release measurements, we analysed the enamel surfaces of the specimens at the end of the experiment, after the last erosion treatment, by scanning electron microscopy (supplemental Fig. 1). Although a total of 4 min erosion in 1% citric acid, pH 3.6, would still classify as rather early and mild erosion, all of them showed the typical honeycomb pattern of eroded enamel. Enamel rods and crystals were visible, but no consistent differences between the groups were observed. This did not allow us to draw any conclusions about differences in the protective effects of the different groups. For comparisons, we also added specimens from an earlier study that had been treated with saliva containing only trace amounts of ions^12^. Contrary to the specimens from the present study, the surfaces of these specimens were still covered to a large degree by the pellicle. This confirms the conclusion from the authors that proteins are able to bind more strongly to the enamel surface if the solution they are in is devoid of ions.

Results from *in vitro* studies are difficult to apply to *in vivo* circumstances and need to be carefully interpreted when trying to explain clinical findings. But while *in vitro* models can never be expected to perfectly simulate the *in vivo* situation, their contribution to the basic understanding of ETW render them important tools. In the present study, the results cannot be directly transferred to the *in vivo* situation, as there are rather large differences between *in vitro* and *in vivo*/*in situ* formed pellicles^28^. Nevertheless, since pellicle formation is a specific process^29^, the basal pellicle layer directly adsorbed to the enamel surface should be quite similar for *in vitro* and *in vivo* pellicles. Therefore, processes happening at the enamel surface should also be comparable for these differently formed pellicles. Because the pellicle formation with the different kinds of saliva used in this study is impossible to be carried out *in vivo*/*in situ*, we chose this *in vitro* model as a proxy.

**Figure 5:**
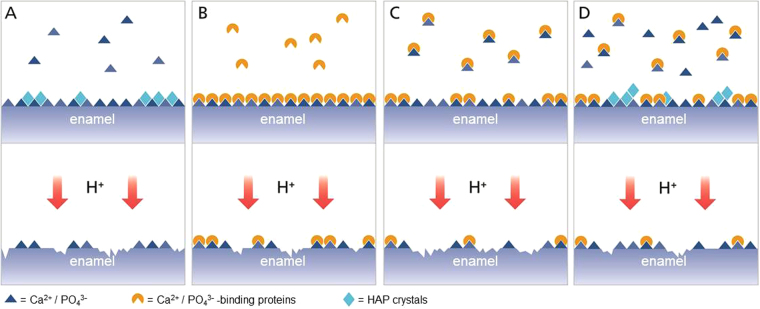
Model of protection of the enamel surface from erosion by different types of saliva. (**A)** Artificial saliva without proteins. **(B)** Dialysed saliva without ions. **(C)** Dialysed saliva with low ion concentrations. **(D)** Dialysed saliva with high ion concentrations.

The results from the present study combined with results from an earlier study^12^ have led to the following model for solutions containing either calcium and phosphate, salivary proteins, or both calcium and phosphate and salivary proteins: In solutions containing calcium and phosphate ions but no proteins, depending on the degree of saturation with respect to enamel, HAP deposits/crystals can form on the surface. These deposits confer some protection to the underlying enamel surface, but they are readily dissolved during subsequent acid attacks (Fig. 5A). In saliva deprived of mineral ions, and thus a solution containing only salivary proteins, the calcium and phosphate binding proteins can bind strongly to the enamel surface. There is no competition for the binding sites by free ions, therefore all those proteins can bind to the surface. Although such a solution is undersaturated with respect to enamel, the protection the proteins confer to the surface is much larger than the demineralizing effect of the undersaturated solution. This leads to an overall strong protective effect of such a solution^12^ (Fig. 5B). In a solution containing salivary proteins and low amounts of calcium and phosphate, some of the ions will bind to the proteins in solution. This, on the one hand, eliminates free ions and can render the solution undersaturated with respect to enamel, on the other hand, it blocks proteins from binding to the surface. In this way, the protective effect of the proteins is vastly reduced. The protective effect of the ions is even reversed into a state of undersaturation, which itself can lead to demineralization of the surface (Fig. 5C). In a solution containing salivary proteins and high amounts of calcium and phosphate, binding of proteins to the enamel surface is again in competition with the binding of free calcium and phosphate ions. However, in contrast to a solution containing low amounts of mineral ions, the binding of free ions does not render the solution undersaturated with respect to enamel. It will still be supersaturated, and HAP deposits/crystals can still form on the surface. In this case, the lower amount of protection conferred by proteins compared to the ion-free solution is partly compensated by the deposits formed on the surface (Fig. 5D). Although the protective effect of such a solution is smaller than the effect of the ion-free solution, it is at least not demineralizing.

## Conclusions

The erosion protection conferred by salivary pellicles depends on an interplay between salivary proteins and salivary ions. Both proteins and ions provide protection on their own, but the degree of protection they provide when combined depends on their relative concentrations. The binding of proteins to calcium on the enamel surface competes with the binding of free calcium in solution, which reduces the proteins available for pellicle formation. On the other hand, the binding of calcium by proteins reduces the concentrations of free ions in solution, rendering the solution undersaturated and unable to deposit minerals onto the enamel. In the worst case, a combination of proteins and ions can even lead to disadvantageous effects, as observed in the present study for the DS_low_ and DS_medium_ groups. Whereas earlier studies showed good protection of enamel by salivary proteins alone, no group containing only proteins was tested in the present study, and the best protection was provided by HS. For AS and DS groups, the best protection was provided with the highest calcium concentrations. This implies that if there is calcium present, it should be at a high concentration to provide protection.

## Electronic supplementary material

Supplementary information
