# Supplementary material for: The effect of different salivary calcium concentrations on the erosion protection conferred by the salivary pellicle
Source: Sci Rep. 2017 Oct 11;7:12999. doi: 10.1038/s41598-017-13367-3 (PMC5636785; doi:10.1038/s41598-017-13367-3)

Supplementary information

The effect of different salivary calcium concentrations on the erosion protection conferred by the salivary pellicle

Baumann T*, Bereiter R, Lussi A, Carvalho TS

Department of Preventive, Restorative and Pediatric Dentistry, University of Bern, Freiburgstrasse 7, CH-3010, Bern, Switzerland.

* Corresponding Author
Tommy Baumann (✉)
Department of Preventive, Restorative and Pediatric Dentistry,
University of Bern, Freiburgstrasse 7, CH-3010, Bern, Switzerland.
Tel.: +41 31 632 86 02
Fax: +41 31 632 98 75
E-mail: tommy.baumann@zmk.unibe.ch

Supplemental materials

Materials and Methods

Scanning electron microscopy

At the end of the last erosion cycle, two specimens from each group were mounted on aluminum stubs and sputtered with gold/palladium (100 s, 50 mA) using a sputtering device. Scanning electron microscopy (SEM) was performed with a JSM-6010 PLUS/LV scanning electron microscope (JEOL, Tokyo, Japan), at 5 kV. SEM images were taken from representative areas with 500× and 2000× magnification factors.

Figures

Supplemental Figure 1. SEM pictures of samples at the end of the experiment, at (A) 500-fold magnification and (B) 2000-fold magnification. Groups are labelled at the top left corner of each picture. The HS/DW group is from Baumann et al.[12] and labelled according to the labelling used in that study. No consistent differences were observed between the groups, except for the HS/DW group that was still largely covered by a pellicle.

Supplemental figure 1

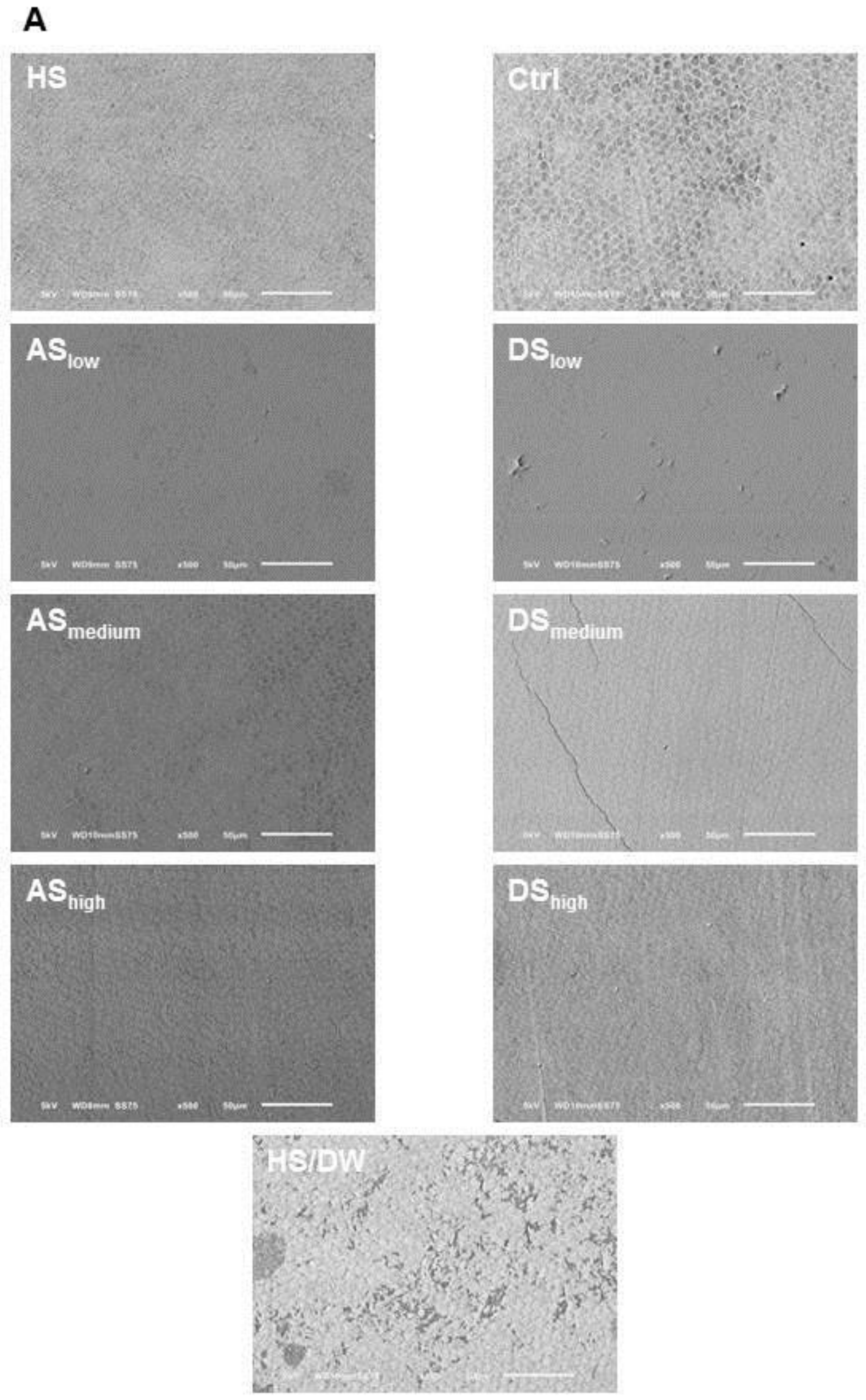

B

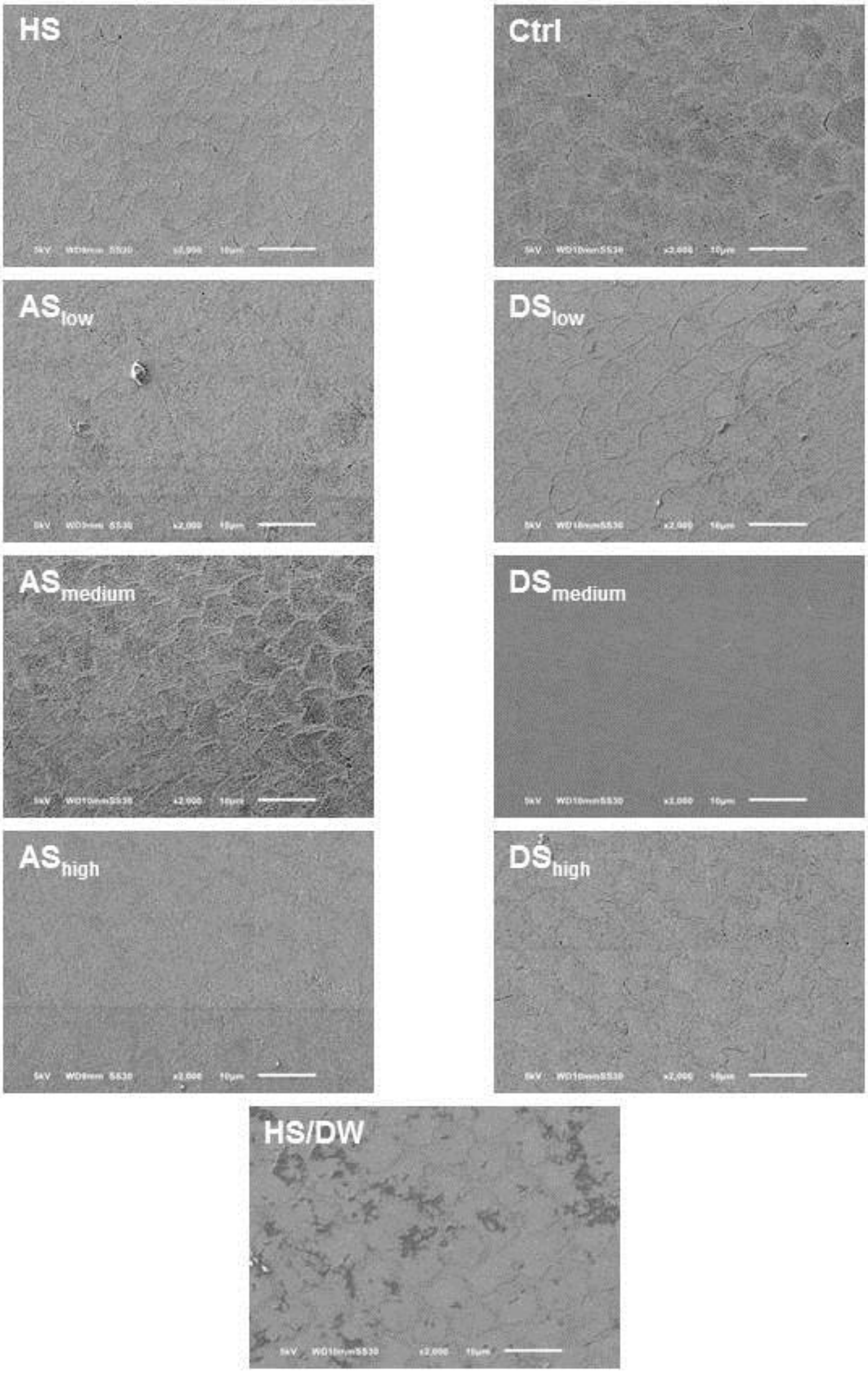

Supplement: Supplementary file 1 — Supplementary information [file 41598_2017_13367_MOESM1_ESM.pdf]
